# Affective Empathy, Theory of Mind and Social Functioning in Patients With Focal Epilepsy

**DOI:** 10.3389/fpsyt.2022.887411

**Published:** 2022-07-11

**Authors:** Birgitta Metternich, Kathrin Wagner, Maximilian J. Geiger, Andreas Schulze-Bonhage, Martin Hirsch, Michael Schönenberg

**Affiliations:** ^1^Department of Neurosurgery, Epilepsy Center, Medical Center - University of Freiburg, Freiburg, Germany; ^2^Department of Clinical Psychology and Psychotherapy, University of Tübingen, Tübingen, Germany

**Keywords:** epilepsy, theory of mind, MASC, empathy, ecological validity, amygdala, frontal lobe, temporal lobe

## Abstract

**Objective:**

Social cognition comprises basic and more complex functions, such as theory of mind (ToM) and affective empathy. Although everyday social interactions may be impaired if such higher-order social cognitive functions are compromised, associations between social functioning and social cognition in people with focal epilepsy (PWFE) are still poorly understood. We used a novel, naturalistic approach to investigate ToM in PWFE by applying the Movie for the Assessment of Social Cognition (MASC). Furthermore, we studied affective empathy, the relationship between social cognitive parameters and measures of social functioning, as well as between epilepsy focus and ToM.

**Methods:**

Thirty patients with either temporal (TLE) or frontal lobe epilepsy (FLE) were compared to 29 healthy control subjects (HC). In addition to the MASC, we applied questionnaire measures assessing empathy and everyday social functioning.

**Results:**

PWFE, especially with FLE, performed significantly worse than HC on the MASC. Perceived social integration and social activities, but not affective empathy, were reduced in PWFE. Regression analyses revealed associations between perceived social integration, clinical group status, affective empathy and ToM.

**Conclusion:**

PWFE displayed ToM deficits during a naturalistic task, whereas affective empathy was unimpaired. FLE may be associated with especially compromised ToM performance. Social cognition and social functioning appear to be interrelated in PWFE, whose self-perceived levels of social integration and social activities are lower than those of HC. More research into the association between social cognition and social functioning in PWFE is needed, in order to develop tailored intervention programs for these patients.

## Highlights

- This study reports the novel application of the MASC, an ecologically valid video-based theory of mind (ToM) task, in patients with focal epilepsy (PWFE).
- During MASC task performance, PWFE showed ToM deficits when compared to healthy control subjects, but no impairment in affective empathy.
- PWFE perceived themselves as lonelier or more poorly socially integrated, and were socially less active than healthy controls.
- Affective empathy and ToM may be associated with perceived social integration in PWFE.

## Introduction

People with focal epilepsy (PWFE) often suffer from cognitive impairment (1), which can significantly reduce their quality of life and limit their participation in social activities (2–4). As a collective, these patients receive less social support, have fewer children, and more often remain single or unemployed (4). While deficits in social cognition may exacerbate these social restrictions (4), little is known about their impact on PWFE. Social cognition comprises basic processes, such as facial emotion recognition, as well as higher-order social functions. The more complex social functions, such as theory of mind (ToM), are the focus of the present study. ToM is defined as the ability to recognize the difference between one's own and another person's mental state, to take on the perspective of that other person, and then infer their thoughts, emotions and intentions (5–7). Possessing a ToM enables a person to predict someone else's behavior (7). Empathy is a much broader term, encompassing cognitive elements, which are needed to form a ToM, as well as affective aspects (7, 8). Affective or emotional empathy is known as the ability to feel what others are feeling (7, 8). The affective state of others leads to an emotional response, such as feeling sad or compassionate for someone who has suffered a loss, or showing an appropriate emotional response to another person's distress (7). Baron-Cohen and Wheelwhright (7) argue that the cognitive and affective aspects of empathy have distinct yet overlapping components, and thereby ascribe ToM to the cognitive component of empathy. Evidence from neuroimaging studies supports two distinct empathy systems: an emotional system (i.e., affective empathy) and a cognitive system comprising ToM in its two forms, cognitive ToM (i.e., inferring thoughts, beliefs and intentions) and affective ToM (i.e., inferring emotions) (8). Imaging studies in healthy subjects have demonstrated ToM performance-associated areas of activation at the temporoparietal junction, as well as in temporal and prefrontal brain regions, and activation within the frontoparietal and limbic networks is associated with affective empathy (8–15). However, in a naturalistic setting, these network processes are likely to co-occur.

PWFE are known to show deficits in ToM (16–18), whereby functional connectivity in crucial brain areas can be compromised ictally and interictally through brain lesions or dysfunctions due to the spreading of seizure activity (19). Possibly, mesiotemporal structures, e.g., the amygdala, play an important role in ToM performance, due to their position in relevant networks (20, 21). Patients whose epilepsy began at an early age may show more marked impairment in ToM than those with a later disease onset (20, 21). Children with epilepsy also show deficits in ToM relative to healthy controls (22), and ToM deficits are potentially related to seizure frequency in both children and adolescents (22, 23). While it may not be feasible to measure ToM and affective empathy separately, some tests favor the measurement of more cognitive processes, while others preferably tap the emotional response to another person's distress. The majority of studies investigating ToM in PWFE have employed the Faux-Pas test (FPT) (24–29), a measure based on written vignettes requiring adequate language skills, which can be compromised in focal epilepsy (30). During the FPT, subjects cannot make use of information from other channels (e.g., facial or vocal expressions, body language) whilst mentalizing, in contrast to everyday social interactions.

The present study thus used a different tool for evaluating ToM, namely the Movie for the Assessment of Social Cognition (MASC) (31), which was originally developed for patients with Asperger's autism (31) and has been employed to investigate social-cognitive deficits in various psychiatric disorders (32, 33). The MASC is a movie depicting everyday social interactions. The questions prompt test subjects to infer emotions, thoughts and intentions of the characters in the film, thereby measuring cognitive as well as affective ToM. Furthermore, the MASC scoring protocol allows for differentiating between various types of ToM errors: Insufficient ToM (undermentalizing) or excessive ToM (overmentalizing). Due to its video-based format allowing for a multi-modal task presentation, the MASC may be more ecologically valid (34) than conventional ToM tasks or other purely verbal tasks. Complex ToM tasks such as the MASC place high demands on verbal and executive functioning (14, 34, 35) (e.g., cognitive flexibility), which can be compromised in TLE and FLE (36). However, even after controlling for executive and verbal abilities, deficits in ToM (as measured with the FPT) have been shown in epilepsy patients (29, 37). Whether this also holds true for ToM as measured with the MASC is unknown to date.

Pathological changes regarding structure and functional connectivity of the amygdala have been identified in patients with autism-spectrum disorders (38, 39), and these patients perform more poorly on the MASC (31). Therefore, MASC deficits are also foreseeable in patients with TLE, especially those with amygdalar damage. In a similar vein, because patients with schizophrenia display frontal lobe pathology (40) and show performance deficits in the MASC compared to healthy controls (33), frontal lobe epilepsy (FLE) might also elicit a subnormal MASC performance. However, few studies have directly compared ToM performance in patients with FLE and TLE (17). Investigations into the type of MASC error in groups of psychiatric patients have revealed differential error profiles; for example, affective disorders seem to be associated with under- rather than overmentalizing (41). Similarly, there might be different MASC error profiles in epilepsy patients, given that temporal (TLE) and frontal lobe epilepsy (FLE) share neurobiological features with affective disorders (42).

Few studies have investigated emotional or affective empathy in epilepsy. In some studies, the application of self-report questionnaires to measure affective empathy did not identify any differences between healthy controls and PWFE (43–46). One study reported a reduction in affective empathy in patients with right-sided TLE when compared to those with left-sided TLE and healthy controls (47).

Although deficits in complex social cognition (ToM and affective empathy) are believed to lead to participation restrictions, such associations have rarely been investigated to date (2, 17). Prior studies have mostly used self-report measures of quality of life (QoL) instead of direct measures of social functioning (29, 48–50). However, it is important to assess social functioning or social integration directly and distinguish it from general QoL as well as epilepsy-related variables. The relationship between everyday social functioning and social cognition in epilepsy also needs to be evaluated with adequate measures (2). In PWFE, the association between social functioning and ToM has not been tested with measures that are presumably more ecologically valid, such as the MASC. In psychiatric research an association between ToM performance on the MASC and deficits in everyday social functioning (e.g., employment and relationship status, social integration) has been found (31, 51, 52). As a consequence, tailored intervention programs have been developed and evaluated for psychiatric patients (53). It would be important to study such associations in PWFE (2, 54). This issue ought to be given high priority considering that the identification of an association between those two fields of functioning can facilitate the development of interventions that strengthen social cognition and, in turn, promote increased social participation in PWFE.

Based on ToM deficits in PWFE previously described in literature (16, 17), we hypothesized that

1. Adult PWFE show deficits in ToM measured with a video-based task simulating everyday experience (MASC) compared to healthy control subjects.

Based on the majority of the few studies investigating affective empathy in PWFE (43–46), we further hypothesized that

2. Affective empathy does not differ between PWFE and healthy control subjects.

Based on the few studies investigating social functioning in PWFE (2, 17, 54), we hypothesized that

3. PWFE are less socially active and less integrated (i.e., lonelier) than healthy controls.

Further *exploratory* analyses were conducted on subgroups of patients addressing the following hypotheses:

1. Both FLE and TLE patients are more impaired in relation to MASC performance than healthy controls. Amygdalar damage in TLE is associated with poorer MASC performance.
2. An association between social cognition and social functioning exists in PWFE.

Furthermore, we were interested in determining how demographic and clinical parameters as well as cognitive performance are each associated with ToM and affective empathy.

## Methods

### Subjects

The present study was approved by the ethics committee of Albert-Ludwigs-University Freiburg. All subjects provided written informed consent. The present study has been conducted in accordance with the Declaration of Helsinki.

Healthy control subjects (HC) without a history of psychiatric or neurological disorders as established through history, MINI and BDI-II (see Instruments) were acquired through advertisements on the Freiburg University Medical Center Intranet pages. Patients and healthy control subjects were included if their verbal IQ was in the normal range (≥85), if they were native Germans or proficient in German and ≥18 years old. Healthy control subjects with BDI-II-scores of >13 were excluded.

Epilepsy patients were excluded if they had BDI-II-scores above 28 and/or they showed (i) severe mood disorders or other severe psychiatric disorders including schizophrenia or neurodevelopmental disorders such as autism-spectrum disorders or ADHD, (ii) other relevant neurological disorders (e.g., neurodegenerative disease), (iii) focal epilepsy originating from regions other than the temporal or frontal lobes, or (iv) if they had received epilepsy surgery.

We recruited in-hospital patients who had undergone presurgical evaluation at the Epilepsy Center, Freiburg University Medical Center. The site of the epilepsy focus was identified by Video-EEG monitoring. All patients were pharmacoresistant. Three patients were excluded due to a severe episode of depression (*n* = 1), idiopathic generalized epilepsy (*n* = 1), and no participation in the MASC (*n* = 1). One control subject was excluded because of an acute adjustment disorder. Clinical data from the 30 enrolled epilepsy patients (TLE subgroup, *n* = 20; FLE subgroup, *n* = 10) are presented in Table 1. Within the TLE subgroup, half the patients had unilateral amygdalar damage or pathology (AmyD), as diagnosed by high-resolution MRI performed in accordance with the routine epilepsy protocol. Table 2 presents the demographic and clinical data from the epilepsy patients vs. healthy controls (control group, *n* = 29) (for data on individual patients see supplementary material).

**Table 1:** Clinical features of the entire patient cohort and subgroups.

	**PWFE**			
	***(n** **=** **30)***			
**Variable**	**TLE**		**FLE**	
	***(n** **=** **20)***		***(n** **=** **10)***	
**Age at epilepsy onset**	***M***	***SD***	***M***	***SD***
	20.3	12.4	24.9	22.6
**Seizure type** ^**1, 2**^	*n*		*n*	
No seizures Focal aware seizures Focal unaware seizures Bilateral tonic-clonic seizures	0 11 16 10		0 5 6 4	
**Focus lateralization**				
Dominant hemisphere Non-dominant hemisphere Bilateral Inconclusive	11 6 2 1		5 3 2 0	
**MRI pathology** ^**1**^				
Unspecific (WML, gliosis, signal alterations) Tumor/Cavernoma Hippocampal sclerosis/atrophy Focal cortical dysplasia Amygdalar hyper-/hypoplasia Encephalocele Cerebral infarction Limbic encephalitis Temporal polar atrophy Contusion defect None	4 6 3 2 6 1 1 1 1 0 1		4 2 1 2 0 0 0 0 0 1 1	
**Medication count**				
Monotherapy Polytherapy No medication	7 12 1		2 8 0	
**Medication (total daily dosage)**	***M***	***SD***	***M***	***SD***
BRV CBZ CNB CLB ESL LCM LEV LTG OXC PER PGB PHT VPA	180 1,200 150 5 1800 365 2916.7 314.3 1,500 6.7 450 - 2,000	20.9 282.8 0 N/A N/A 234.3 801.0 193.5 N/A 6.4 N/A - N/A	220 1200 - 15 1,000 400 2500.0 335.7 - 12.0 400 300 300	57.0 N/A - N/A N/A N/A 353.6 190.9 - N/A N/A N/A N/A

*BRV, brivaracetam; CBZ, carbamazepine; CNB, cenobamate; CLB, clobazam; ESL, eslicarbazepine; FLE, frontal lobe epilepsy; LCM, lacosamide; LEV, levetiracetam; LTG, lamotrigine; OXC,oxcarbazepine; PER, perampanel; PGB, pregabalin; PHT, phenytoin; PWFE, People with focal epilepsy; TLE, temporal lobe epilepsy; VPA, valproic acid; WML, white matter lesions.*

*^1^Multiple classification possible.*

*^2^Seizures during the previous two months*.

**Table 2:** Demographic parameters and cognitive measures for epilepsy patients versus control subjects.

**Variable**	**PWFE**		**Healthy controls**		***p***	**Cohen's d/w**
	**(*****n*** **=** **30)**		**(*****n*** **=** **29)**			
	***n***		***n***			
**Sex**						
Female	16		21		0.18	0.2
Male	14		8			
	***M***	***SD***	***M***	***SD***		
Age	38.2	13.9	35.6	11.0	0.544	0.2
Verbal IQ (MWT-B)	105.9	12.2	112.3	14.1	0.053	−0.5
Verbal short-term memory (digits forwards, WMS-R score)	7.1	2.0	7.8	1.8	0.162	−0.4
Verbal working memory (digits backwards, WMS-R score)	6.3	1.3	7.1	1.8	0.057	−0.5
Cognitive Flexibility (sec.) (TMT-B-TMT-A)	42.3	22.4	35.6	19.2	0.174	0.3

*FLE, frontal lobe epilepsy; MWT-B, Mehrfachwahlwortschatztest (Version B); PWFE, People with focal epilepsy; TLE, temporal lobe epilepsy; TMT, Trail Making Test (higher values correspond to worse performance); WMS-R, Wechsler Memory Scale Revised*.

## Measures

### History

We briefly interviewed each participant to acquire information about neurological/psychiatric disease history, handedness, and demographics such as employment and relationship status.

### Psychiatric Disorders

The Mini International Neuropsychiatric Interview [MINI (55)], a short structured interview for assessing the major psychiatric axis-I disorders according to DSM-IV and ICD-10, was carried out in all control subjects and all patients who did not receive a psychiatric consultation during their presurgical evaluation. Furthermore, all subjects completed the Beck Depression Inventory, 2nd edition [BDI-II (56, 57)]. Subjects are asked to rate on a four-point scale (0–3) the occurrence of 21 symptoms, e.g., sadness: “I do not feel sad”; “I feel sad much of the time”; “I feel sad all the time”; “I am so sad or unhappy that I can't stand it.”

### Affective Empathy

Affective empathy was measured using the Toronto Empathy Questionnaire [TEQ (58)].

Sixteen Items Are Rated on a Five-Point Likert Scale (0-4), e.g., “When Someone Else Is Feeling Excited, I Tend to get Excited too”. High Values Indicate a High Level of Affective Empathy.

### Theory of Mind

ToM abilities were assessed using the Movie for the Assessment of Social Cognition [MASC; (31)]. The movie portrays a dinner party attended by four people. Following each movie sequence, subjects are prompted to answer a question with four response choices. Each question relates to the assumed mental states of one of the characters in the movie. As complex mental states are concerned, the task allows uncovering subtle ToM deficits as well as more pronounced deficits. There are two main categories of erroneous responses: (1) *undermentalizing (ToM- errors):* (a) either a complete lack of ToM or (b) insufficient recognition of mental states, (2) *overmentalizing (ToM*+ *errors)*: excessive presumption of mental states. All errors are added together for a total MASC sum score, which can range between zero and 45. “MASC errors” will refer to the sum score from hereon, unless otherwise stated.

### Social Functioning

The degree of social activity was measured with the Social Activity Log [SAL (59)]. Subjects are asked to rate how often they perform certain social activities, e.g., “In the past month, circle a number for how many times you: Had family or friends come to visit.” (0–6 or more). The 16 SAL questions do not refer to seizure parameters, but assess social activities independently. Another important aspect of social functioning pertains to how lonely and isolated vs. how well-socially integrated someone feels. For this purpose we used the revised UCLA Loneliness Scale (60), which consists of 20 items with a five-point Likert scale (1–4), e.g., “How often do you feel alone?”. High values correspond to heightened loneliness or poor social integration. The questions do not refer to epilepsy-related parameters.

### Cognitive Tests

A brief neuropsychological test battery was used in the present study. We chose instruments that are routinely applied at our Epilepsy Center for presurgical neuropsychological assessment: Verbal intelligence was estimated with a vocabulary test, the Mehrfachwahl-Wortschatz-Intelligenztest (MWT-B) (61). Subjects are asked to recognize and mark each genuine German word in rows of five. In accordance with the *multiple choice* principle, each line contains a word that is known colloquially or in academic language among four fictitious new constructions. In order to assess executive functioning and short-term/working memory in a time-efficient manner, we included the following parameters: (i) cognitive flexibility, which was measured with the Trail-Making Test (TMT) (62) and defined as the difference between TMT-B and TMT-A, (ii) short-term and working memory, which were assessed by the forward and backward digit spans derived from the Wechsler Memory Scale [WMS-R (63)].

### Statistical Analyses

The chi-squared (χ^2^)-test was used to compare sex and focus lateralization distribution between the groups. Mann-Whitney-U tests were applied to compare demographic measures, parameters of social functioning and general cognition between the groups, as well as in order to determine the association of focus localization and amygdalar pathology with ToM. In order to test for the association of verbal IQ, cognitive flexibility and short-term/working memory with group differences in complex social cognition, we conducted analyses of covariance (ANCOVA). Spearman's rank or Pearsons correlations were conducted to explore the association between ToM/affective empathy and clinical and demographic parameters. Exploratory linear regression analyses (method enter) were conducted to identify predictors of social functioning. In order to meet the conditions for conducting a regression analysis, UCLA scores were converted into normal scores. The standardized residuals were then normally distributed. Associations between variables were linear and homoscedasticity was present. All analyses were conducted with IBM SPSS Statistics 27 (64).

## Results

### Demographic, Clinical, and Cognitive Parameters

The PWFE group did not differ significantly in age or sex from healthy control subjects (see Table 2). Patient subgroups did not differ from each other in age, age at epilepsy onset, sex, focus lateralization, cognitive parameters or number of anticonvulsant drugs, nor did the patient subgroups differ from healthy controls in age, sex or verbal IQ (χ^2^-test and Mann-Whitney-U test, all *p*-values > 0.1). In the TLE group 10 patients showed amydalar pathology. Mean group performance on cognitive measures is presented in Table 2. There were no significant differences between PFWE and controls in estimated verbal intelligence or other cognitive parameters. However, verbal intelligence (*p* = 0.053) and verbal working memory (*p* = 0.057) trended toward statistical significance in favor of the control group.

### MASC Performance in PWFE Compared to Healthy Control Subjects

Patients made significantly more ToM errors (MASC total errors) than healthy controls (*p* = 0.002). Further analyses on MASC error type (ToM+ and ToM- errors) yielded no significant group differences for ToM+ (*p* = 0.092), but for ToM- (*p* = 0.035) (see Table 3). The latter difference was reduced to a statistical trend following Bonferroni correction. An ANCOVA controlling for verbal IQ as well as executive functioning (cognitive flexibility, working memory) revealed a significant effect of group (F = 5.3, *p* = 0.026) on overall MASC errors, while working memory (F = 0.3, *p* = 0.60) showed no significant association with the result. However, verbal IQ and cognitive flexibility were significantly associated with MASC errors (F = 4.7, *p* = 0.034, and F = 5.3, *p* = 0.026, respectively).

**Table 3:** Measures of social functioning and social cognition.

**Variable**	**PWFE**		**Healthy**		***p^1, 2^***	**Cohen's *d***
			**controls**			
	**(*****n*** **=** **30)**		**(*****n*** **=** **29)**			
	***M***	***SD***	***M***	***SD***		
Social activities (SAL)	33.4	12.8	40.1	9.6	**0.020**	−0.6
Social integration (UCLA loneliness scale)	36.5	12.5	27.2	5.0	**0.002**	1.0
Affective empathy (TEQ)	47.6	6.3	49.4	5.6	0.60	−0.3
ToM (MASC errors)	14.2	5.3	10.2	3.8	**0.002**	0.9
Overmentalizing	5.8	2.9	4.6	2.6	0.092	0.5
(MASC ToM+ errors)						
Undermentalizing	8.4	5.2	5.6	3.1	***0.035***	0.7
(MASC ToM- errors)						

*MASC, Movie for the Assessment of Social Cognition; PWFE, People with focal epilepsy; SAL, Social Activity Log; TEQ, Toronto Empathy Questionnaire; UCLA, loneliness scale: high values correspond to elevated loneliness/low social integration.*

*^1^**bold** = survived Bonferroni correction.*

*^2^**bold and italics** = statistical trend after Bonferroni correction (p ≤ 0.1)*.

### Univariate Correlations Between Clinical/Demographic Parameters and ToM

Neither gender, sex, current age, age at epilepsy onset, disease duration nor the number of anticonvulsive medications were significantly correlated with ToM (MASC errors). Out of all the cognitive parameters assessed, only verbal IQ and cognitive flexibility were significantly correlated with ToM (*r* = −0.38, *p* = 0.003, *r* = 0.35, *p* = 0.009).

### Affective Empathy in PWFE Versus Healthy Control Subjects

Self-assessed affective empathy (TEQ) did not differ significantly between groups (*p* = 0.60) (see Table 3). Within the ANCOVA verbal IQ and working memory as well as cognitive flexibility did not display a significant association with affective empathy (F = 0.02, *p* = 0.884; F = 2.4, *p* = 0.13; F = 0.02, *p* = 0.889, respectively).

### Univariate Correlations Between Clinical/Demographic Parameters and Affective Empathy

Neither current age, age at epilepsy onset, disease duration nor the number of anticonvulsive medications were significantly correlated with affective empathy (TEQ). Across the entire study cohort, sex was significantly correlated with affective empathy, with women showing higher scores (greater affective empathy) than men (*r* = −0.42, *p* = 0.001). Verbal IQ, working memory and cognitive flexibility were not significantly correlated with affective empathy.

### Exploratory Analyses of Patient Subgroups

#### MASC Performance and Affective Empathy in FLE Patients, TLE Patients and Healthy Controls Association of MASC Performance With Amygdalar Damage

**Figure 1:**
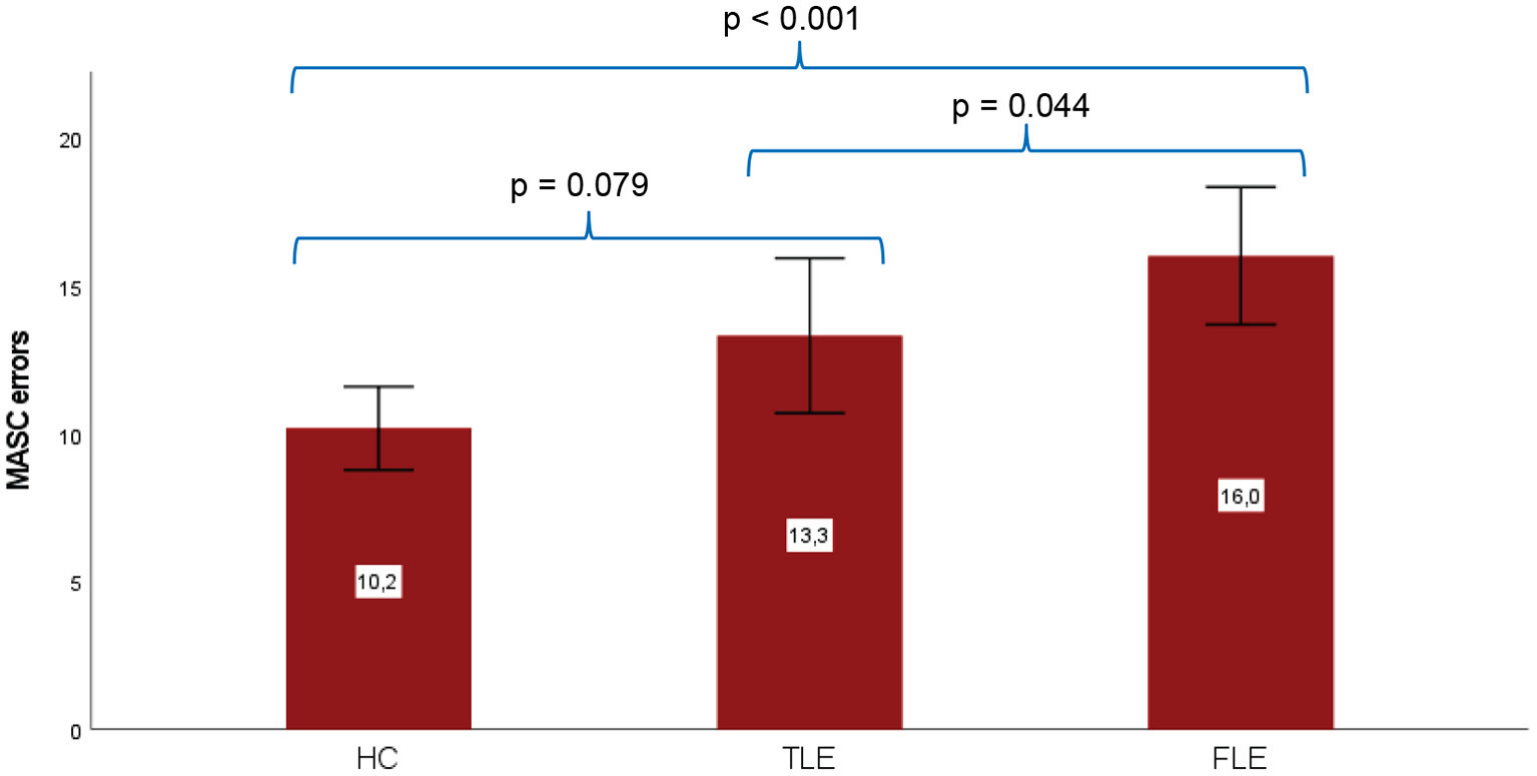
ToM performance (MASC errors: higher values correspond to worse performance, mean values ± 2 standard errors shown on each bar) according to group status. Healthy controls (*N* = 29), TLE patients (*N* = 20) and FLE patients (*N* = 10). MASC, Movie for the Assessment of Social Cognition. Significance levels for subgroup differences are shown prior to Bonferroni correction.

Patients with TLE and FLE showed a statistically significant difference on ToM performance (see Table 4) with FLE performing worse than TLE (*p* = 0.044), which, however, did not survive alpha correction (according to Bonferroni). When both groups were compared to the control group, TLE showed a statistical trend toward impaired ToM performance (*p* = 0.079), which did not survive Bonferroni correction either, whereas the FLE group performed significantly worse (*p* < 0.001), and this difference survived Bonferroni correction (see Table 4, Figure 1). Figure 1 shows subgroup comparisons of MASC performance in TLE and FLE patients. An ANCOVA controlling for verbal IQ as well as executive functioning (cognitive flexibility, working memory) showed a statistically significant effect of focus group (F = 6.5, *p* = 0.003) on overall MASC errors, while working memory (F = 0.5, *p* = 0.50) was not significantly associated with the outcome. However, verbal IQ and cognitive flexibility were significantly associated with MASC errors (F = 4.9, *p* = 0.031, and F = 5.6, *p* = 0.022, respectively). Affective empathy (TEQ) did not differ significantly in any of the subgroup comparisons (see Table 5). A further ANCOVA revealed that verbal IQ and working memory as well as cognitive flexibility did not display a significant association with affective empathy (F = 0.02, *p* = 0.902; F = 2.4, *p* = 0.13; F = 0.02, *p* = 0.883, respectively). Finally, in order to explore a potential association between amygdalar pathology in TLE and ToM performance, we compared TLE patients with and without AmyD to healthy controls. MASC performance in the TLE group with AmyD was significantly worse compared to that in the control group (mean 15.2, SD 6.4, d = 1.1, *p* = 0.015, surviving Bonferroni correction), whereas in TLE patients without AmyD, it was not (mean 11.4, SD 4.8, d = 0.3, *p* = 0.74).

**Table 4:** ToM performance according to group status (epileptogenic focus, control group).

**Patient subgroups**	**MASC errors**		***p^**1, 2**^***	**Cohen's *d***
	***M***	***SD***		
**Localization**				
TLE (*n* = 20)	13.3	5.9	0.044	−0.5
FLE (*n* = 10)	16.0	3.7		
HC (*n* = 29)	10.2	3.8	0.079^a^/**<** **0.001**^b^	0.7^a^/1.5^b^

*FLE, frontal lobe epilepsy; HC, Healthy controls; MASC, Movie for the Assessment of Social Cognition; TEQ, Toronto Empathy Questionnaire; TLE, temporal lobe epilepsy.*

*^1^p-values are shown prior to Bonferroni correction.*

*^2^**bold** = survived Bonferroni correction.*

*^a^TLE vs. HC.*

*^b^FLE vs. HC*.

**Table 5:** Affective empathy according to group status (epileptogenic focus, control group).

**Patient subgroups**	**TEQ**		***p^1^***	**Cohen's *d***
	***M***	***SD***		
**Localization**				
TLE (*n* = 20)	47.3	6.5	0.664	−0.1
FLE (*n* = 10)	48.1	6.2		
HC (*n* = 29)	49.4	5.6	0.428^a^/0.813^b^	−0.4^a^/−0.2^b^

*FLE, frontal lobe epilepsy; HC, Healthy controls; TEQ, Toronto Empathy Questionnaire; TLE, temporal lobe epilepsy.*

*^a^TLE vs. HC.*

*^b^FLE vs. HC.*

*^1^p-values are shown prior to Bonferroni correction*.

### Social Functioning (Loneliness/Social Integration and Social Activities) in PWFE and Healthy Controls

Social functioning operationalized as self-assessed social integration (UCLA loneliness scale) and social activities (social activities questionnaire, SAL) were significantly reduced in epilepsy patients compared to healthy controls (*p* = 0.002 and *p* = 0.02, respectively, see Table 3), i.e., PWFE were less socially active and at the same time felt lonelier or less well-integrated than control subjects. These differences remained significant after alpha-correction (Bonferroni).

### Association Between Social Cognition (ToM or Affective Empathy) and Social Functioning

We conducted an exploratory linear regression analyses (method: enter) of the entire study cohort. The following independent variables were entered into the model: MASC errors, affective empathy (TEQ) and group status (patients vs. controls). The normal scores from the UCLA loneliness scale were entered as dependent variable. The final model (corrected *R*^2^ = 0.31) included group status (beta = −0.32, *p* = 0.01) and affective empathy (beta=-0.38, *p* = 0.002). Being in the control group as well as having higher affective empathy ratings were associated with lower UCLA scores, and hence a higher level of self-assessed social integration. A further exploratory regression analysis on self-assessed social integration was conducted within the PFWE group, in order to identify statistical predictors of UCLA loneliness normal scores that are independent of group status. Independent variables entered into the model were MASC errors and affective empathy (TEQ). The final model (corrected *R*^2^ = 0.42) included affective empathy (beta = −0.48, *p* = 0.005) and MASC errors (beta = 0.37, *p* = 0.023). Higher affective empathy ratings and fewer MASC errors were associated with lower UCLA scores, and hence a higher level of self-assessed social integration. The same two regression analyses were repeated with social activity (SAL) used as the dependent variable. None of the independent variables entered into the model reached statistical significance.

## Discussion

### The Cognitive Component of Empathy: ToM as Measured With a Naturalistic Task (MASC)

In the present study ToM performance in subgroups of patients with focal epilepsy (PWFE) was evaluated with the MASC, a naturalistic video-based measure of social cognition. Only two studies in the field have previously used a video-based task to measure ToM (11, 65), which comprised a test battery of video vignettes displaying either basic emotional states, or interactions based on sarcasm and deception. These studies found deficits in PWFE. In terms of complex social cognitive inferences, unlike the tests used in these earlier studies, the MASC is not confined to the recognition of basic emotional states, sarcasm and deception, but simulates real-life interactions amongst a small group of people. The first question we aimed to address was whether PWFE as a group show impaired MASC performance (total MASC errors) compared to healthy controls. Our results show that this is indeed the case, which is in line with previous studies reporting the detection of ToM deficits in PWFE using other types of tasks (18, 26, 27, 29). The group difference in ToM performance persisted after controlling for verbal IQ, cognitive flexibility and working memory, which is concordant with previous research findings based on other types of ToM tasks (29, 37, 50). Giovagnoli et al. (29) showed that in a factor analysis, ToM as measured with the Faux-Pas (FPT) task loaded on a factor separate from other neuropsychological measures, such as TMT-A/-B, short-term/working memory or verbal fluency. In our analyses we observed significant correlations between verbal IQ, executive functions and ToM, as well as a statistically significant association between verbal IQ and MASC scores in the ANCOVA. However, when verbal IQ and executive functioning were controlled for, there was still a significant MASC impairment in the PWFE group. Hence, in agreement with previous findings (21, 29, 50), we argue that ToM impairment in the MASC cannot be fully explained by general cognitive deficits in PWFE. Although correlations between cognitive variables and social cognition in epilepsy have also been reported in previous research (26, 66), social cognitive deficits in epilepsy appear to represent a distinct impairment, which could be explained by epilepsy-related dysfunction in brain areas implicated in basic emotion recognition or theory of mind (19). Since FLE and TLE share neurobiological pathologies with affective disorders (42), like patients with major depression (41), people with FLE and TLE may commit more undermentalizing errors on the MASC compared to HC. Our results appear to support this notion, even though the difference in undermentalizing did not survive Bonferroni correction.

Regarding epileptogenic focus and overall ToM performance, Bujarski et al. (65) found that the location of the epileptic focus did not significantly influence the results when patient subgroups were directly compared. Our study demonstrated that FLE patients displayed worse ToM performance compared to both TLE patients and controls (although the former did not survive Bonferroni correction). In accordance with this finding, a systematic review (17) found that although effect sizes for ToM deficits were large in both FLE and TLE patients, the effect size for FLE was descriptively larger. The entire TLE group showed a mere trend toward poorer ToM scores compared to the control group. However, within the TLE group patients with amygdalar pathology may be relatively more impaired in ToM task performance. Some evidence exists for ToM deficits in adult patients with mTLE (17, 20, 21). Yet, these studies did not compare groups of TLE patients with healthy and pathological amygdalae. Shaw et al. (20) compared patients with unilateral amygdalar damage to a heterogeneous clinical control group with focal epilepsy. Especially in patients with early onset amygdalar damage, performance was poorer than in the clinical comparison group. In a study with very small subsamples, children with TLE and unilateral amygdalar resection showed deficits when performing a ToM storybook task (37). Our exploratory analysis finding that amygdalar damage may be associated with worse ToM performance is in line with the literature describing the amygdalae as important structures regarding ToM (9, 10, 13, 47, 67). This may be due to their prominent connections, e.g., to the ventromedial prefrontal cortex and the entire default mode network, which have been linked to ToM in brain imaging studies (8, 68). Focal epilepsy affects networks crucial to social cognition, as the epileptogenic zone may overlap them and alter their functioning (68). Yet, in PWFE, reorganization of (social) cognitive functions may occur, e.g., driven by the epilepsy itself, but also in the aftermath of surgical procedures aiming at the removal of the epileptogenic zone or after other types of brain lesions. Intra- and interhemispheric reorganization processes and their limitations may explain why some patients or groups of patients with epilepsy show more impaired social cognitive functioning than others (69), and, on the other hand, why in some cases no group differences in performance can be detected (70).

Our sample size did not allow for comparing patient subgroups in terms of focus lateralization. Nevertheless, the subgroups did not differ significantly regarding focus lateralization. Lateralization of the epileptogenic focus may play an important role in ToM performance. However, so far it remains undecided whether patients with right TLE show inferior ToM skills compared to patients with left TLE (17, 67). For some social cognitive functions, e.g., recognition of emotional prosody, evidence of lateralization exists (68–70). However, imaging studies have demonstrated reorganization processes and plasticity in PWFE, possibly via recruitment of contralateral cortical areas (70). Then again, it is possible that interhemispheric communication is essential to unimpaired functioning, meaning that both hemispheres may contribute differentially to ensure normal functioning (67, 69). The fact that reorganization of social cognitive functions depends on various individual factors, such as seizures, epilepsy onset and others, might explain why studies on lateralization of ToM have shown inconsistent results (17).

### Affective Empathy as Measured Using the TEQ

Application of the TEQ in present study did not reveal any evidence for a reduction in affective or emotional empathy in PWFE compared to healthy controls. This is consistent with the majority of earlier findings (43–46). One other study did find abnormalities in affective empathy in patients with right-sided TLE when they were compared to patients with left-sided TLE and healthy controls (47). Due to the heterogeneity of our sample regarding epileptogenic focus we were unable to assess the effect of focus lateralization on social cognition in a meaningful manner. We did not find any significant subgroup differences in TEQ ratings, suggesting that focus localization may not be associated with affective empathy. Past research suggests distinct neural networks for affective empathy and cognitive theory of mind (8), which could partly explain this finding.

### Association Between ToM/Affective Empathy and Everyday Social Functioning in PWFE

Knowledge about the degree to which deficits in social cognition affect social functioning in PWFE is of high relevance. Yet, social functioning is difficult to operationalize. On the one hand, objective parameters such as employment or marital status do exist, but these obviously depend on a considerably greater number of influential factors than social cognitive functioning. This especially holds true for epilepsy patients, where epilepsy-related factors may interfere, e.g., with attaining higher education, employment, a driving license etc. Such factors include cognitive deficits, comorbid psychiatric disorders and vulnerability to stress, disease-related factors (such as seizure type and frequency, seizure origin, brain abnormalities, developmental problems, effects of anticonvulsant medication), and last, but not least stigma (3). Therefore, softer indicators of real-life social functioning may be more helpful for studying the relationship between complex social cognition and parameters of social functioning. Such softer indicators of real-life social functioning include self-reported social integration or social activities, which are the parameters we chose to operationalize social functioning in the present investigation. Self-report measures are unfortunately prone to bias. On the other hand, the degree to which a person reports to be socially integrated or socially active, probably comes closest to the individual experience. Therefore, it can serve as an important indicator of participation and well-being in society.

Our results show that PWFE feel lonelier and describe themselves as less socially active than healthy control subjects. The exploratory regression analyses showed that self-reported loneliness or social integration was related to both, group status (patients vs. controls) and affective empathy (TEQ), in the entire sample, and to affective empathy and ToM (MASC errors) in our PWFE group. Social activities, on the other hand, were not significantly associated with ToM or affective empathy. It may therefore be the case that self-reported social activities are highly dependent on seizure parameters in PWFE, thus obscuring a possible association with other variables. A further reason for the non-significant correlations could be the small sample size. Nevertheless, there are first indicators of a possible connection between affective empathy and ToM performance, and one aspect of social functioning, namely self-reported social integration. Correspondingly, other studies have found an association between ToM performance and quality of life (29, 50). Moreover, Wang et al. (25) found an association between the Social and Occupational Functioning Scale for Epilepsy (SOFSE) (57) and the FPT.

### Associations Between ToM/Affective Empathy and Clinical and Demographic Parameters

No significant correlations or even statistical trends emerged for the correlations between ToM (MASC) or affective empathy (TEQ) and current age, age at epilepsy onset, disease duration or drug-load (number of anticonvulsant drugs). Anticonvulsant medication is known to have a potential impact on cognitive functioning [for review see e.g., Eddy et al. (71)], but it does not seem to have affected the current results, since in addition to the lack of significant correlations there were no significant group differences concerning drug-load. Furthermore, no significant correlation emerged between sex and ToM. However, a highly significant correlation between affective empathy and sex across the entire study sample indicated that women had higher affective empathy scores than men. This is in line with previous research into affective empathy showing that females generally achieve higher scores on various measures of affective empathy (72, 73). Nevertheless, this finding also has no relevant impact on the results reported here, since the groups did not differ significantly in the distribution of males and females, nor did they differ regarding affective empathy.

### Limitations and Future Directions

The present results should be interpreted with caution due to the moderate sample sizes. For example, this might be the reason why the difference in ToM (MASC errors) between TLE patients and healthy controls failed to reach significance, or why no significant correlations emerged between age of epilepsy onset and social cognition. Moreover, we only assessed two aspects of social functioning. Therefore, further potential indicators of social functioning need to be explored in future studies applying regression analyses to larger samples. Furthermore, studies with larger samples could help explore the contributions of social cognition [amongst other epilepsy-related factors (3)] on both subjective and objective parameters of social functioning. Our study design does not allow for differentiating between the impact of the social sequelae of seizures and the impact of the seizures themselves on social cognitive performance. Therefore, future studies should try to employ more sophisticated statistical models in order to explore the nature of the association between complex social cognition and social functioning. The well-known limitations of self-report questionnaires also apply to the TEQ, the UCLA loneliness scale and the Social Activity Log. Additional measures of affective empathy and social functioning, that are less prone to social desirability, are needed in future studies. The MASC also has some limitations, such as being sensitive to IQ and executive functions as well as to ToM (34). Furthermore, the presence of contextual cues could mask deficits (34). However, both aspects also contribute to the ecological validity of the MASC, whereas its observer perspective (as opposed to a lifelike self-referent perspective, where one is involved directly in social interactions or at least imagining to be) does not (74). The patient sample included in the present investigation is heterogeneous in terms of epilepsy etiology. Not only patients in the entire PWFE group, but also those in the FLE and TLE subgroups showed brain abnormalities such as tumors, dysplasia and signal alterations. This high degree of heterogeneity therefore makes it impossible to explore the effect of specific etiologies of epilepsy on our study cohort. However, this holds true for many investigations in epilepsy research. Epilepsy patients are in fact a heterogeneous group, and studying just one set of patients with a specific etiology leads to reduced generalizability, despite the advantage of being able to draw more specific conclusions. Previous research lends support to the notion that, e.g., patients with bilateral amygdalar damage or amygdalar damage in the non-dominant hemisphere may especially be impaired in basic or more complex social cognitive tasks (47, 67, 75, 76). The effect of amygdalar pathology on ToM needs to be explored in studies with larger subsamples. Moreover, as the amygdala shows bidirectional connectivity with various brain regions that may be important for social cognition, such as the temporal pole (15, 76) or the ventromedial prefrontal cortex (8), research into neural connectivity during ToM tasks is required, particularly in the context of epilepsy as a network disorder (68, 69). Furthermore, the association between ToM performance and the onset of amygdalar pathology should also be explored in future studies. Given the findings reported by Toller et al. (47), studies with larger subgroups should further investigate affective empathy in relation to the epileptogenic focus, regarding both actual lateralization and hemispheric dominance. Finally, due to the uncertain nature of seizure frequency variables obtained from patient history, we were not able to examine the relationship between seizure frequency and affective empathy or ToM, which has previously been postulated (22, 23). This important aspect should be investigated with the help of digital seizure diaries or seizure frequency data from monitoring devices.

## Conclusion

The results of the present investigation show that PWFE display performance impairment in the MASC, an ecologically valid ToM task, even after controlling for IQ and executive functioning. In our sample, patients with a frontal epileptogenic focus were especially impaired and performed significantly worse than healthy controls. Moreover, TLE patients with damage to the amygdala possibly have a higher degree of ToM-related impairment than healthy controls. Finally, we have been able to show that in our study sample of PWFE, an important aspect of social functioning – namely, self-reported social integration – is associated with affective empathy and ToM. Due to the moderate size of our main sample and the relatively small subgroup sizes, these results need to be replicated in future studies with larger and more homogeneous samples. Research into the nature of social cognitive deficits in epilepsy, as well as their association with everyday social functioning in particular, is essential for the development of effective intervention programs.

## Data Availability Statement

The raw data supporting the conclusions of this article will be made available by the authors, without undue reservation.

## Ethics Statement

The studies involving human participants were reviewed and approved by Ethics Committee of Albert-Ludwigs-University Freiburg. The patients/participants provided their written informed consent to participate in this study.

## Author Contributions

All authors listed have made a substantial, direct, and intellectual contribution to the work and approved it for publication.

## Funding

This study was funded by a research grant from the Research Committee of Albert-Ludwigs-University Freiburg, Germany. The article processing charge was funded by the Baden-Württemberg Ministry of Science, Research and Art and the University of Freiburg in the funding programme Open Access Publishing.

## Conflict of Interest

The authors declare that the research was conducted in the absence of any commercial or financial relationships that could be construed as a potential conflict of interest.

## Publisher's Note

All claims expressed in this article are solely those of the authors and do not necessarily represent those of their affiliated organizations, or those of the publisher, the editors and the reviewers. Any product that may be evaluated in this article, or claim that may be made by its manufacturer, is not guaranteed or endorsed by the publisher.
