# Supplementary material for: Affective Empathy, Theory of Mind and Social Functioning in Patients With Focal Epilepsy
Source: Front Psychiatry. 2022 Jul 11;13:887411. doi: 10.3389/fpsyt.2022.887411 (PMC9309689; doi:10.3389/fpsyt.2022.887411)
Supplement: Supplementary file 1 [file Data_Sheet_1.PDF]

Supplementary material

Table S1

Clinical and demographic parameters for individual patients

Pat no.	Sex	Age	Age at onset	Epilepsy focus[1]	Focus lateralization	Seizure type[2]	MRI pathology[3]	Amygdalar pathology[3]
1	male	31	19	FLE	dominant	FAS, FUAS	Frontal cavernoma	no
2	male	31	11	FLE	dominant	FUAS, atypical absences	Atrophy of fornix and HC	no
3	female	54	50	FLE	dominant	FUAS motor seizures	Frontal FCD	no
4	female	52	9	FLE	dominant	FAS motor seizures, FUAS, BTCS	Unspecific signal alterations	no
5	female	20	18	FLE	dominant	FAS	Frontal gliosis	no
6	female	45	8	FLE	non-dominant	FAS	Frontal FCD	no
7	female	34	4	FLE	non-dominant	FAS, BTCS, atonic seizures	none	no
8	female	52	51	FLE	non-dominant	BTCS	Hypoxic brain damage	no
9	female	35	11	FLE	bilateral	FUAS, BTCS	WML	no
10	male	70	68	FLE	bilateral	FUAS motor seizures	Frontal contusion defect, cavernoma	no
11	male	42	26	TLE	dominant	FAS, FUAS, BTCS	WML	no
12	female	51	47	TLE	dominant	BTCS	Temporal tumor WHO III/IV	yes
13	male	42	1	TLE	dominant	FUAS	HC sclerosis	no
14	female	26	10	TLE	dominant	FAS, FUAS	Amygdalar hyperplasia	yes
15	female	31	26	TLE	dominant	FUAS	none	no
16	female	26	7	TLE	dominant	FUAS, BTCS	Temporal FCD	no
17	female	35	13	TLE	dominant	FAS, FUAS, BTCS	WML	yes

18	female	23	9	TLE	dominant	FUAS	Temporal-polar hypoplasia, possible frontal FCD	no
19	female	36	6	TLE	dominant	FAS, FUAS	HC sclerosis, amygdalar hypoplasia	yes
20	male	60	22	TLE	dominant	FAS, BTCS	Temporal tumor WHO I/II	no
21	male	21	15	TLE	dominant	FUAS, BTCS	Amygdalar hyperplasia	yes
22	male	58	12	TLE	non-dominant	FAS, FUAS, BTCS	Cerebral infarction, HC sclerosis, amygdalar hypoplasia	yes
23	male	51	42	TLE	non-dominant	FAS	Temporal Tumor WHO I/II	yes
24	male	24	22	TLE	non-dominant	FUAS	Temporal cavernoma, amygdalar hyperplasia	yes
25	male	56	34	TLE	non-dominant	FAS, FUAS	Temporal cavernoma	no
26	female	18	16	TLE	non-dominant	FAS, BTCS,	WML	no
27	male	37	31	TLE	non-dominant	FAS, FUAS, BTCS	Temporal tumor WHO I/II	yes
28	female	37	33	TLE	bilateral	FAS, FUAS	Limbic encephalitis, amygdalar hyperplasia	yes
29	male	21	19	TLE	bilateral	FUAS	Temporal encephaloceles	no
30	male	26	14	TLE	unbekannt	FUAS, BTCS	Unspecific signal alterations	no

Note. BTCS: Bilateral tonic-clonic seizures, FAS: focal aware seizures, FUAS: focal unaware seizures, HC: Hippocampus, WML: White matter lesions.

[1]: Established per Video-EEG monitoring.

[2]: Seizures during the previous two months.

[3]: Diagnosed by high-resolution MRI.
